# Allicin Promoted Reducing Effect of Garlic Powder through Acrylamide Formation Stage

**DOI:** 10.3390/foods11162394

**Published:** 2022-08-10

**Authors:** Xiude Li, Wendi Teng, Guangmin Liu, Fengyu Guo, Hanzhu Xing, Yahui Zhu, Jinwang Li

**Affiliations:** 1 School of Food and Health, Beijing Technology and Business University, Beijing 100048, China; 2 Key Laboratory of Functional Dairy, Ministry of Education, Department of Nutrition and Health, China Agricultural University, No. 17 Qinghua East Road, Haidian District, Beijing 100083, China; 3 Institute of Agri-Food Processing and Nutrition, Beijing Academy of Agricultural and Forestry Sciences, Beijing 100097, China; 4 School of Food Science and Engineering, Qilu University of Technology (Shandong Academy of Sciences), Jinan 250353, China

**Keywords:** acrylamide, Maillard reaction, garlic powder, allicin, kinetics evaluation

## Abstract

Background: Acrylamide is formed during food heating and is neurotoxic to animals and potentially carcinogenic to humans. It is important to reduce acrylamide content during food processing. Researchers have suggested that garlic powder could reduce acrylamide content, but the key substance and acrylamide reduction pathway of garlic powder was unclear. Methods: The inhibitory effect of garlic powder on acrylamide in asparagine/glucose solution and a fried potato model system were firstly evaluated. Furthermore, the effect of allicin on the amount of produced acrylamide in the asparagine/glucose solution model system and fried potatoes was studied with kinetic analysis. Results: The freeze-dried garlic powder had a higher inhibition rate (41.0%) than oven-dried garlic powder (maximum inhibition rate was 37.3%), and allicin had a 71.3% attribution to the reduction of acrylamide content. Moreover, the inhibition rate of allicin had a nonlinear relationship with the addition level increase. The kinetic analysis indicated that garlic powder and allicin could reduce acrylamide content through the AA formation stage, but not the decomposition stage. Conclusions: Allicin was the key component of garlic powder in reducing acrylamide content during acrylamide formation stage. This research could provide a new method to reduce acrylamide content during food processing and expand the application area of garlic.

## 1. Introduction

In industry, acrylamide (AA) is usually used to produce polyacrylamide for application in wastewater treatment [1]. However, neurotoxicity and genotoxicity of AA to animals and potential carcinogenicity to humans had been demonstrated [2], and AA is classified as a probable human carcinogen by the International Agency for Research on Cancer (IARC) [3]. Research indicated that AA was usually produced in heat-treated carbohydrate-rich foods such as crisps, potato chips, grain and bread [4,5]. Meanwhile, dietary AA exposure had become a public health concern due to its wide distribution in food [6,7,8]. At the same time, controlling the formation of AA during food processing in heat-treated carbohydrate-rich foods had become of great significance for human health.

In recent years, numerous studies associated with AA have focused on the formation mechanism. The primary mechanism of AA formation was the Maillard reaction between asparagine and reducing sugars at temperatures between 140 °C and 180 °C [9,10,11]. Based on studies of the mechanism of AA formation, extensive methods, including optimization of heating conditions (e.g., heating temperature, heating time and heating method) and selection of suitable food materials or adding exogenous additives, have been reported to reduce AA content in food processing [9,12]. Some food additives have been reported to reduce the formation of AA in food processing, such as amino acids, antioxidants, proteins and melt salts [13].

The addition of natural antioxidants was another way to effectively reduce AA content in food processing. Zhang et al. found that extracts of bamboo leaves (AOB) and green tea (EGT) could effectively inhibit AA formation, and the reduction rates were 74.40% and 74.30%, respectively [14]. Oral et al. reported that extracts of European cranberry bush juice, olive mill wastewater and pomegranate peel had a significant effect on the formation of AA [15].

On the other hand, Garlic (*Allium sativum*) was well known for its anti-inflammatory, antifungal, and antiseptic properties [16]. Modern medicine has found that garlic had potential medical value, which included lowering blood pressure, and had a certain preventive effect on cardiovascular and cerebrovascular diseases, antioxidant and anti-aging, antitumor, and antiviral activities, advancing the function of the liver and improving glucose metabolism and antibacterial function [17,18,19]. Researchers also suggested that the main bioactive compounds of garlic were thio-sulfinate compounds, especially diallyl thio-sulfonate (allicin) and S-allyl-cysteine sulfoxide (alliin) [20]. Therefore, it is important to analyze the thio-sulfinate content when evaluating the active function of garlic. Furthermore, allicin represents about 70% of the active components in garlic and could provide an antioxidant function to the body due to its sulfonyl [21,22,23]. Yuan et al. found that allicin could effectively reduce AA content in an asparagine/fructose model system, and the inhibition rates exceeded 50% [24]. Li et al. reported that garlic powder significantly inhibited AA formation, and the reduction rate was 43% [25]. Makarim et al. suggested that garlic could significantly decrease the AA content in bread [26].

However, garlic powder was a mixed substance. It was difficult to clarify the key substance reducing AA content. In order to identify the main active substance of garlic powder that contributes to its reducing effect on AA content, the glucose/asparagine solution model system and fried potato were selected as research models for study. Meanwhile, the kinetic analysis method was used to evaluate the AA reduction pathway of garlic powder and allicin. To the best of our knowledge, this research was the first study to focus on the main active substance of garlic powder in reducing AA content in heated food. Through research, the application of garlic powder and allicin in the food field could be further extended to provide new ideas for healthy food production.

## 2. Materials and Methods

### 2.1. Materials and Chemicals

Fresh Jinxiang garlic was provided by Sunmoon Food Company (Tai’an, China). Fresh potato was purchased from a local supermarket in Tai’an (Tai’an, China). Acrylamide (98%) was purchased from Meryer Chemical Technology Co., Ltd. (Shanghai, China). Asparagine (Asn, 99%) was obtained from Capitabio Co., Ltd. (Shanghai, China). Glucose (Glc, 99%) was obtained from Kaitong Chemical Technology Co., Ltd. (Tianjin, China). Chromatography grade methanol was supplied by Shandong Yuwang Industrial Co., Ltd. (Dezhou, China). Doubly deionized water (DDW) was obtained from a Water Pro water system (Labconco Corp., Kansas City, MO). Other chemicals used in this work were analytical grade, and all solutions were prepared in DDW.

### 2.2. Design of the Experiment

Considering that the production process could change the content of the biologically active substance in garlic powder, especially allicin, in this research different production processes (freeze-dried (FD) and oven-dried in different temperatures) were first used to prepare the garlic powder, in order to clarify the effect of garlic powder on the reduction of AA content. Subsequently, the garlic powder was added to a Glc/Asn solution system or fried potato system for detection of AA content reduction. In order to further clarify which main substances in garlic powder could reduce AA content, the thio-sulfinate content of garlic powder was detected, for thio-sulfinate is the main biologically active component of garlic [27]. Thus, the allicin was further added into a Glc/Asn solution system for detection of AA content reduction [20]. Meanwhile, in order to expound the AA reduction strategy for garlic powder and allicin, a kinetic analysis method was used. Finally, the main substances of garlic powder in AA content reduction were clarified in detail.

### 2.3. AA Detection

AA analysis was developed according to the method of Xu Longhua [28]. A Shimadzu (Shimadzu, Kyoto, Japan) 2010 LC equipped with two LC-10ATVP pumps and a Shimadzu SPD-10AVP ultraviolet detector were utilized. An analytical reversed-phase Thermo C18 column (4.6 mm × 250 mm; Agela Technology, Tianjin, China) was used to achieve all separations at a mobile phase flow rate of 0.7 mL/min. The mobile phase was methanol/water (2:98, *v*/*v*), and the oven temperature was 30 °C. The injection volume was 5 μL, and the detection was operated at 205 nm. CLASS-VP software was used to acquire and process spectral and chromatographic data.

### 2.4. Detection of the Influence of Garlic Powder Production Process on Thiosulfinate Content

#### 2.4.1. Preparation of FD Garlic Powder

Fresh Jinxiang garlic bulbs were cut into 4 mm slices, rolled out and placed at −20 °C to freeze for 24 h. The pre-frozen garlic slices were freeze-dried for 24 h and ground to powder, and the powder was sieved with a 60-mesh sieve.

#### 2.4.2. Preparation of Oven-Dried Garlic Powder

Fresh Jinxiang garlic bulbs were cut into 4 mm slices and then rolled out and placed in an oven at 40 °C, 45 °C, 50 °C, 55 °C or 60 °C to oven dry. When the garlic slices arrived at a constant weight, they were ground to powder and sieved with a 60-mesh sieve.

#### 2.4.3. Detection of Thio-Sulfinate Content of Garlic Powder

Thio-sulfinate analysis was performed according to the method of Lawson [29], in which 1 g garlic powder was weighed into 15 mL DDW. After shaking for 1 min, the mixture was placed for 9 min at room temperature and centrifuged in 4 °C for 5 min, then 1 mL of supernatant was mixed with 5 mL L-cysteine (10 mmol/L), and 1 mL of reaction mixture was diluted to 100 mL using DDW. Then, 4.5 mL of the reaction mixture was reacted with 0.5 mL of DTNB (5,5’-Dithiobis-(2-nitrobenzoic acid), 1.5 mmol/L) at 26 °C for 15 min, and the absorbance was measured at 412 nm by ultraviolet spectrophotometer.

### 2.5. Detection of Reducing Effect of Garlic Powder on AA Content in Glc/Asn Solution Model System

To study the effect of garlic powder on AA formation in the model system, 1.2 mmol of glucose, 1.2 mmol of asparagine, and 0.05 g garlic powder (FD garlic powder and oven-dried garlic powder) were weighed separately into an autoclave, and then 2 mL DDW was added. After shaking in a vortex mixer for 0.5 min, the mixture was heated in an oil bath at 190 °C for 30 min (the optimum formation conditions of AA). After heating, the mixture was immediately removed and cooled to room temperature. After filtration through a 0.22 µm filter membrane, the filtrate was injected into the HPLC for analysis.

### 2.6. Detection of Reducing Effect of Garlic Powder on AA Content in Fried Potato Chips

#### 2.6.1. Influence of Immersion Time on AA Content in Fried Potato Chips

In order to evaluate the inhibition effect of garlic powder on potato chips, the potato chips were soaked in FD garlic powder for different times. 50 g of FD garlic powder was added to 500 mL of DDW. Potato chips (35 × 10 × 10 mm) were added to the above mixed liquor at a solid-liquid ratio of 1:4 and immersed for 30 min, 60 min, 90 min, 120 min or 150 min in ultrasonic conditions. The control group was placed in DDW for 30 min, 60 min, 90 min, 120 min or 150 min under the same conditions. The soaked potato chips were removed and washed with DDW to remove the surface garlic powder solution, and the surface moisture was absorbed with bibulous paper. Then, the immersed potato chips were fried at 160 °C, 180 °C, and 200 °C for 20 min, the time when acrylamide content was the highest. After the surface oil of the fried potato chips drained off, they were dried and ground to powder for later use.

2 g of fried potatoes was accurately weighed and mixed with 18 mL DDW and 5 mL n-hexane. After mixing in the vortex mixer for 2 min, the mixture was layered. The supernatant was extracted, and 10 mL of acetonitrile, 4 g of MgSO_4_ and 1 g of NaCl were added. After vibration for 1 min, the mixed liquor was centrifuged at 4 °C for 10 min (10,000 r/min), and 8 mL of acetonitrile layer was added to a 50 °C water bath, mixed with nitrogen, dissolved in 1 mL chromatographic pure methanol and filtered with a 0.22 µm filter for analysis.

#### 2.6.2. Kinetic Study of the Reducing Effect of Garlic Powder on AA Content in Fried Potato Chips

The same batch of potato strips was divided into two portions. In one, as the control system, the potato strips were soaked in DDW for 120 min at 25 °C. In the other, potato strips were soaked for 120 min at 25 °C with FD garlic powder solution (100 g/L). Potato trips were drained before being fried. Due to the fact that the AA content of fried potato did not decrease after frying for 60 min, a frying time was chosen between 5 min and 60 min. Peanut oil was preheated to 200 °C, and potato strips were fried for 5 min, 10 min, 15 min, 20 min, 25 min, 30 min, 35 min, 40 min, 45 min, 50 min, 55 min and 60 min. After being fried, potato strips were cooled down to room temperature immediately, and peanut oil adhering to potato strips was removed.

### 2.7. Detection of Reducing Effect of Allicin on AA Content in Glc/Asn Solution Model System

#### 2.7.1. Extraction of Allicin

The extraction method was performed according to Songsungkan with some modifications [30]. Allicin was extracted from fresh Jinxiang garlic. The coating of fresh garlic was peeled off and crushed by 6.5 g mortar. The garlic paste was incubated in a water bath at 38 °C for 20 min. Then, diethyl ether was added at a ratio of 1:4 (g/mL), and the mixture was incubated in a water bath at 28 °C for 30 min and then centrifuged (10,000 r/min) for 10 min to remove the residue. The supernatant of diethyl ether was removed by a rotary evaporator at 30 °C. When all of the diethyl ether was evaporated, allicin was dissolved in methanol to 50 mL.

#### 2.7.2. Allicin Concentration Detection

The allicin solution (10 mL) was put into a triangular flask, then 10 mL of Hg(NO_3_)_2_ (0.1 mol/L) were added. Shake well and stand to complete the reaction, then add 20 mL of HNO_3_ (8 mol/L). Then add DDW to 100 mL, add 2 mL of NH_4_Fe(SO_4_)_2_ (0.75 mol/L), and titrate with KSCN (0.1 mol/L) until it becomes slightly orange-red and forms flocculent precipitation as the end point. Recording the number of milliliters consumed, the concentration of allicin was 219.1 mg/L. The quantitative analysis was based on the following formula.

$$\mathrm{Allicin}\;\mathrm{concentration}=\;\frac{M_{1\;}\times {\;V}_{1}-0.5\times {\;M}_{2\;}\times {\;V}_{2}}{V_{0}}\;\;\times \;0.05409$$

In this formula:

M_1_ was standard solution concentration of Hg(NO_3_)_2_; 0.1 mol/L;

V_1_ was consumed volume of Hg(NO_3_)_2_; standard solution, mL;

M_2_ was standard solution concentration of KSCN; 0.1 mol/L;

V_2_ was consumed volume of KSCN; standard solution, mL;

V_0_ was volume of allicin solution, 10 mL.

0.05409 was the mass of allicin which equivalent to 1 mmol/L of Hg(NO_3_)_2_;

#### 2.7.3. Determination of the Effect of Allicin on AA Content Reduction

According to the experimental method and steps of evaluation of the inhibiting effect of garlic powder on AA content, 50 μL, 100 μL, or 150 μL allicin solutions dissolved in methanol and ethanol, respectively, were added to the Glc/Asn solution model to study the effect of different solvents on the inhibition of AA.

According to the above method, 50 μL of 10.95 mg/L, 21.91 mg/L, 43.82 mg/L or 219.1 mg/L allicin solutions were added to the Glc/Asn solution system to study the effect of different additive amounts of allicin on the inhibition of AA content.

Twenty microliters, 30 μL, 40 μL, 50 μL, 60 μL, 70 μL, 80 μL, 90 μL or 100 μL of allicin solution were added to the Glc/Asn solution system. The other steps were the same, in order to study the effect of different volumes of allicin on the inhibition of AA content.

#### 2.7.4. Dynamic Research of Inhibition of Allicin against AA

Allicin solution (219.1 mg/L, 50 μL) was added to the Glc/Asn solution model, and then diluted to 2 mL with DDW. The solution was heated at 190 °C for 0 min, 10 min, 20 min, 30 min, 40 min, 50 min or 60 min.

### 2.8. Choice of Kinetic Model

Many studies had shown that the formation of AA in food can be described by the following process: K*_F_* and K*_E_* represent the formation and elimination rate constants of AA, respectively [31].

$$\mathrm{Glc}+\mathrm{Asn}\;\overset{K_{F}}{\to }\;\mathrm{AA}\;\overset{K_{E}}{\to }\;\mathrm{AA}\;\mathrm{elimination}\;\mathrm{products}$$

Based on this process, three kinetic models were established to describe the formation and degradation of AA: the logistic–Fermi kinetic model, logistic–exponential kinetic model and first-order formation/elimination model, and through comprehensive comparisons, kinetic models explaining AA production and degradation were identified [32,33,34]. Many studies had shown that the first-order formation/elimination model and logistic–Fermi kinetic model were not suitable to describe the kinetics of AA reduction formation in Glc/Asn solution systems [14,25,35]. The first-order formation/elimination model had two rate constants, K*_F_* and K*_E_*, to describe the formation and elimination of AA. However, this kinetic model had a poor correlation with experimental AA content data, and parameter K*_F_* did not match well with the formation stage, so the first-order formation/elimination model was not suitable [25]. The logistic–Fermi kinetic model had five parameters, including a, k_g_ (min^−1^), t_cg_ (min), k_d_ (min^−1^) and t_cd_ (min); therefore, it was more flexible than the first-order formation/elimination model [32,33]. In the logistic–Fermi kinetic model, the parameters a, k_g_ and t_cg_ were temperature-dependent coefficients during the AA formation stage, and k_d_ and t_cd_ were temperature-dependent coefficients during the AA elimination stage. Both Zhang and Li et al. found that the Logistic–Fermi kinetic model had a better pseudo-R^2^ and its fitness also seemed good, but the values of t_cd_ in the fitted models did not match the Logistic-Fermi kinetic model due to t_cd_ < 0. Meanwhile, Zhang and Li et al. found that garlic powder could reduce AA content through inhibition of AA formation, and not AA digestion. Compared with the results of Zhang and Li et al., our kinetic study of allicin’s effect on AA content in Glc/Asn solution system also found the inhibition effect on the AA formation stage, and this is the same for Zhang and Li et al. [25]. Therefore, the logistic–Fermi kinetic model could not be used in this study.

The logistic–exponential kinetic model seemed to be the best choice to describe AA formation and elimination. The Logistic–Exponential kinetic model (Equation (1)) describes the formation of AA by a logistic function (Equation (2)) and degradation by an exponential function (Equation (3)) as follows:

C(t) = Cg (t)·Cd (t)(1)

(2) $$\mathrm{Cg}\;(t)=\frac{a}{1+\exp[\mathrm{kg}(\mathrm{tcg}-t)]}-\frac{a}{1+\exp(\mathrm{kg}\cdot \mathrm{tcg})}$$

Cd (t) = exp (−t/τ)(3)

In these equations, C(t) represents the change in AA content in the whole processing, Cg (t) represents the concentration of AA formed, and Cd (t) represents the concentration of AA eliminated. a, k_g_ and t_cg_ were temperature-dependent coefficients during the AA formation stage, and τ is a parameter related to time.

### 2.9. Data Analysis

The effect of garlic powder and allicin on the Glc/Asn solution system was evaluated by t-test, and analysis of differences among kinetic parameters was performed by Duncan’s multiple range tests. SAS software (SAS Institute, Cary, NC) was used to perform significance testing for all the data.

The kinetic model was evaluated by the pseudo-R^2^ [36] as follows:

$$\mathrm{Pseudo}-R^{2}=1-\frac{\mathrm{SS}(\mathrm{residual})}{\mathrm{SS}(\mathrm{corrected})}$$

where SS(residual) and SS(corrected) were the residual sum of squares and corrected sum of squares, respectively, and both were obtained by SAS software.

## 3. Results and Discussions

### 3.1. Effect of Garlic Powder Production Process on Thio-Sulfinate Content

Many studies have demonstrated that the main active substance in garlic were thio-sulfinates, and eight kinds of thiosulfinate components, including allicin, had similar structures (R-S-S-(O)-R) [35]. The majority of the biological activities of garlic were sulfur-containing compounds, which were formed from alliin under the reaction of alliinase [37,38].

**Figure 1:**
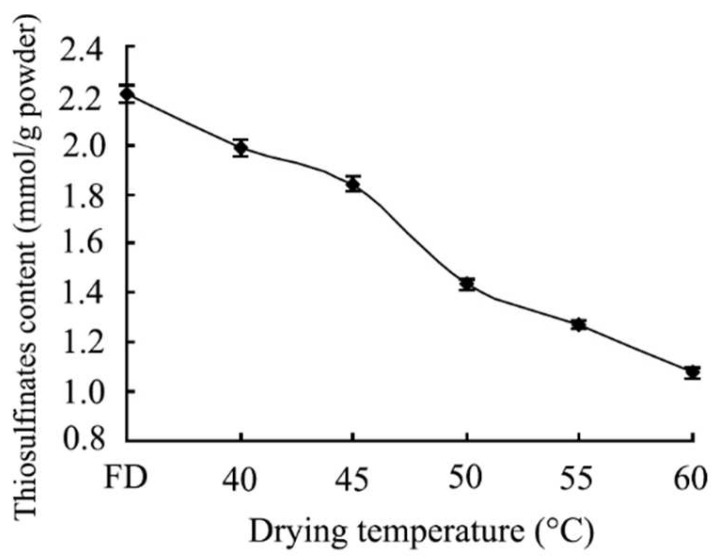
Effect of drying temperature on thiosulfinate content in garlic powder. FD (Freeze dried garlic powder).

As shown in Figure 1, the content of thio-sulfinates in garlic powder prepared by the oven drying method decreased slowly with increasing drying temperature. When the temperature was at 40 °C, the content of thio-sulfinates was 1.99 mmol/g, which is the maximum content in the oven drying method. Meanwhile, the content of thio-sulfinates showed a downward trend after 40 °C until it reached 1.1 mmol/g at 60 °C

The results might be related to alliin enzyme activity, for thio-sulfinates could be converted from alliin by alliin enzyme. Alliin enzyme had different activities when garlic powder was prepared at different temperatures by the oven drying method, which led to the formation of different contents of thio-sulfinates. Because alliin enzyme had a better effect on thiosulfinate formation at 40 °C, it could be inferred that the optimum temperature of the alliin enzyme was 40 °C (Figure 1). On the other hand, thio-sulfinates decomposed faster at higher temperatures, which might be due to higher temperatures accelerating the decomposition of thio-sulfinates [39].

On the other hand, compared with garlic powder prepared by the freeze-drying method (FD garlic powder), the content of thio-sulfinates in garlic powder prepared by the oven drying method was lower, and the content of thio-sulfinates in FD garlic powder reached 2.2 mmol/g. Before FD garlic powder was prepared, garlic slices were prefrozen for 24 h at −20 °C, so that it was easy for FD garlic powder to form enough thio-sulfinates, and difficult to decompose thio-sulfinates under a low temperature environment (Figure 1).

### 3.2. Effect of Garlic Powder Production Process on AA Inhibition Rate of Glc/Asn Solution System

Casado et al. found that blanched garlic could significantly reduce AA content in olive and had no significant effect on olive firmness and color [40]. Meanwhile, since thio-sulfinates were known as the main ingredients of the active substances in garlic powder, they might be considered as the main factor in reducing AA content in heat-treated carbohydrate-rich foods.

**Figure 2:**
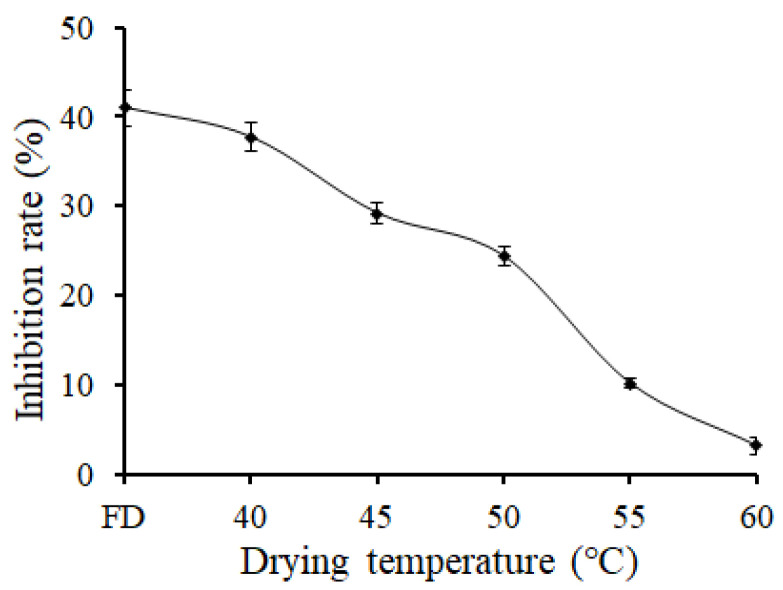
Effect of garlic powder drying temperatures on the inhibition rate of acrylamide in Glc/Asn solution system.

In order to clarify the effect of thio-sulfinates on AA content reduction, the inhibition rate with different drying temperatures of garlic powder on AA formation in the Glc/Asn solution system was analyzed (Figure 2). The inhibition rate of garlic powder on AA content decreased along with an increase in garlic powder heating temperature, and the inhibition rate trend of garlic powder on AA content was the same as thio-sulfinate content. The inhibition rate reached 41.0% and 38.6% when garlic powder was prepared by the freeze-drying method and oven drying method at 40 °C, respectively. This might be due to the thiosulfinate compound reducing the AA content by a Michael-type reaction, for thio-sulfinates contain nucleophilic groups, so that thiosulfinate compound attack the electrophilic carbon of the vinyl group of AA by its nucleophilic groups [41,42].

### 3.3. Effect of FD Garlic Powder on AA Inhibition Rate of Fried Potato Chips

**Figure 3:**
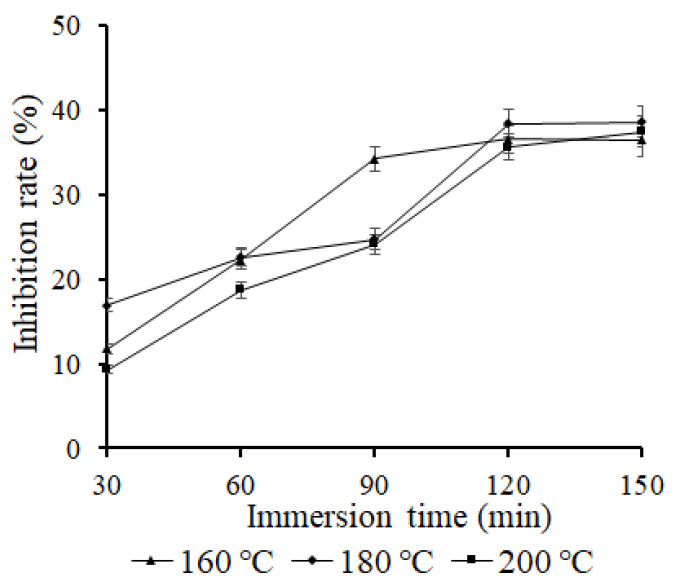
Effect of immersion time on acrylamide inhibition rate in fried potato chips.

In order to clarify the inhibition effect of FD garlic powder on the AA content of fried potato chips, these contents were evaluated under different frying temperatures (Figure 3). Meanwhile, in order to analyze the reduction effect of FD garlic powder, the frying time was chosen as 20 min, when AA content of fried potato was highest. The results suggested that the reducing effect of garlic powder on the AA content of fried potato chips increased along with increase in immersion time. When the immersion time exceeded 120 min, the changes in the inhibition rate of AA content did not increase. The diffusion effect of active ingredients in garlic grew greatly due to the action of ultrasound, and more active ingredients were absorbed into potato chips with increasing immersion time, which increased the inhibition rate of AA content in potato chips. When the immersion times were 120 min and 150 min, the changes in the inhibition rate were not obvious, so an immersion time of 120 min was selected in this test for further study.

### 3.4. Kinetics Evaluation of Garlic Powder on AA Content in Fried Potato Chips

**Figure 4:**
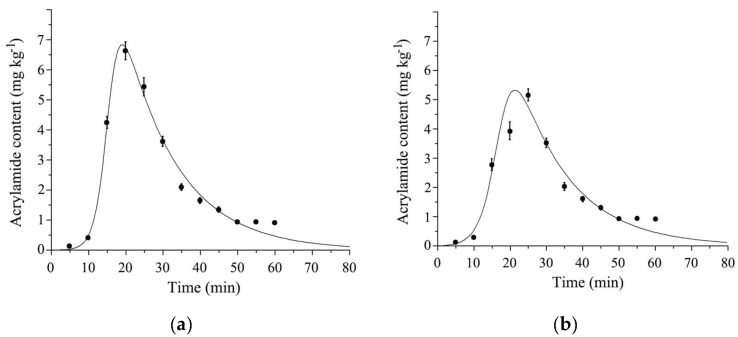
Logistic−exponential kinetic model fitting curves for acrylamide contents in garlic powder. (**a**) AA content of fried potato without garlic powder soaking treatment; (**b**) AA content of fried potato with garlic powder soaking treatment.

By using SAS software and Marquadrt NLS as the fitting technology, the data were processed to establish the kinetic model and calculate kinetic parameters. The fitted curves of the logistic-exponential model are shown in Figure 4a,b. The results showed that the experimental values were uniformly distributed around the fitted curve. Therefore, it could be determined that there was a strong correlation between the model’s predicted values and experimental values of the control or experimental group.

The logistic-exponential parameters are shown in Table 1. The R^2^ of the logistic exponential was calculated and was more than 0.98, which indicated that this model had a good fitting effect and could be used for dynamic research. The data obtained according to Duncan’s multiple comparison method showed that the related parameters of the AA formation stage were obviously different from those of the control group. However, the related parameters of the digestion stage were not significantly different from those of the control group. The results indicated that the inhibition of FD garlic powder on AA content was embedded in the AA formation stage and the thio-sulfinates of garlic powder contributed to the AA content reduction [43,44].

### 3.5. Effect of Allicin on AA Content in Glc/Asn Solution System

#### 3.5.1. Effect of Allicin on AA Inhibition Rate

**Figure 5:**
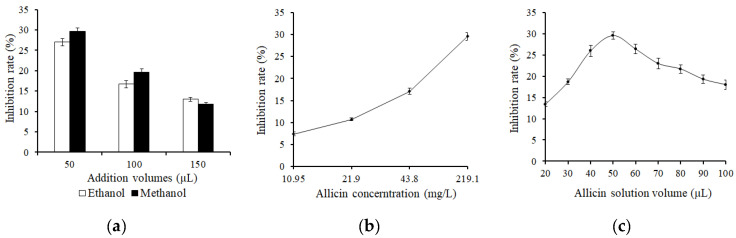
Reducing effect of allicin on acrylamide content of Glc/Asn solution system. (**a**) Effect of reconstitution solvents of allicin on acrylamide inhibition rate; (**b**) Effect of allicin concentration on acrylamide inhibition rate; (**c**) Effect of allicin addition volumes on acrylamide inhibition rate.

As shown in Figure 5a, the inhibition rate with different solvents of allicin solution on AA formation was studied. Under the addition of 50 μL and 100 μL, the inhibition rate using methanol as the solvent was significantly higher than that using ethanol as the solvent. Therefore, methanol was chosen as the solvent for the study of the inhibitory effect of allicin on AA content.

As shown in Figure 5b, the inhibition rate of AA gradually increased as the concentration of allicin increased. When the concentration of allicin was 219.1 mg/L, the inhibition rate was obviously higher than that of the other concentrations. Therefore, a concentration of 219.1 mg/L was chosen for the later study.

The effect of different volumes of allicin on the inhibition of AA content was studied. As shown in Figure 5c, the best inhibition rate was obtained when the addition volume of allicin solution was 50 μL, which was 29.6%. Therefore, 50 μL was selected as the optimal addition volume of allicin solution for further study.

#### 3.5.2. Kinetics Evaluation of Allicin Reducing AA Content

**Figure 6:**
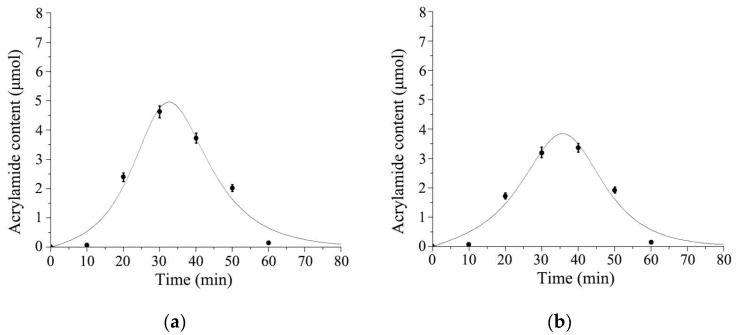
Logistic-exponential kinetic model fitting curves for acrylamide contents in allicin. (**a**) AA content without garlic powder soaking treatment; (**b**) AA content with garlic powder soaking treatment.

The fitted curves of the logistic-exponential model are shown in Figure 6a,b. The results showed that the experimental values were uniformly distributed around the fitted curve. Therefore, it could be determined that there was a strong correlation between the model’s predicted values and experimental values of the control or experimental group.

The logistic-exponential parameters are shown in Table 2. The R^2^ of the logistic exponential model was more than 0.98, which indicated that this model also had a good fitting effect for dynamic research on the inhibition of AA content by allicin. The data obtained according to Duncan’s multiple comparison method showed that the related parameters of the AA formation stage (k_g_, t_cg_) were obviously different from those of the experimental group. However, the related parameters of the digestion stage had no significant change. The results indicated that the inhibition of allicin on AA content played an important role in the AA formation stage, which was the same as the result of the inhibition of FD garlic powder on AA content in fried potato chips. This might be due to the sulfur-base of allicin contributing to the reduction effect of garlic powder through a Michael-type reaction, for allicin is the main thio-sulfinate compound and biologically active component of garlic [41,45]. On the other hand, the antioxidant property of allicin might also contribute to the AA content inhibition, for allicin could scavenge the chain-carrying peroxyl radicals by the transfer of the allylic hydrogen atom (α-sulfenyl proton) to form hydroperoxides, then inhibit the reaction of amino acid and glucose, and decrease Schiff base formation [46,47].

## 4. Conclusions

Garlic is an easily accessible food and numerous studies have shown that allicin was the main biologically active substance. In this research, allicin was proved to be the main substance in garlic powder in reducing AA content. In addition, allicin could not promote the elimination stage, but inhibited the formation stage to reduce AA content. This research is helpful for suggesting a more rational use of the effective ingredients in garlic to lower AA content. It will provide new ideas for the application of garlic powder and allicin in healthy food production.

## Figures and Tables

**Table 1:** Model discrimination results for the logistic-exponential kinetic model in fried potato chips.

Group	*a*	*k_g_* (min^−1^)	*t_cg_* (min)	*τ* (min)	Pseudo-*R*^2^
Control	28.5 ± 2.2	0.6 ± 0.0	15.6 ± 0.1	14.6 ± 0.6	0.99
Garlic powder	26.6 ± 0.7	0.4 ± 0.0 *	17.4 ± 0.1 *	14.8 ± 0.2	0.99

Data for the kinetic parameter presentation were expressed as the mean ± SD, and the value of the same parameter with * indicates a significant difference at the 95% confidence interval compared with the control system.

**Table 2:** Model discrimination results for the logistic-exponential kinetic model in Glc/Asn solution system.

Group	*a*	*k_g_* (min^−1^)	*t_cg_* (min)	*τ* (min)	Pseudo-*R*^2^
Control	147.6 ± 17. 5	0.2 ± 0.0	30.4 ± 0.4	10.9 ± 1.9	0.98
Allicin	401.4 ± 74.4 *	0.2 ± 0.0 *	36.2 ± 0.7 *	9.5 ± 0.4	0.98

Data for the kinetic parameter presentation are expressed as the mean ± SD, and the value of the same parameter with * indicates a significant difference at the 95% confidence interval compared with the control system.

## Data Availability

The data presented in this study are available on request from the corresponding author.
